# An Extremely Rare Case of Streptococcus anginosus Bacteremia Associated With Colon Cancer and Infective Endocarditis

**DOI:** 10.7759/cureus.9368

**Published:** 2020-07-24

**Authors:** Chandrakala Dadeboyina, Salman Khan, Litty Thomas

**Affiliations:** 1 Internal Medicine, Guthrie Clinic/Robert Packer Hospital, Sayre, USA

**Keywords:** streptococcus anginosus colon cancer, streptococus anginosus endocarditis

## Abstract

Streptococcus anginosus (S. anginosus) is a subgroup of viridans streptococci that tend to form a deep-seated abscess. These bacteria can be part of healthy human flora and commonly found in the gastrointestinal tract and oral cavity. Infective endocarditis is most commonly caused by Staphylococcus aureus and Streptococcus. Among the Streptococcus group, anginosus is extremely rare to cause endocarditis, and there are only a few case reports available. We present a patient with S. anginosus bacteremia who subsequently got diagnosed with metastatic colon cancer along with aortic and mitral valve endocarditis

## Introduction

Endocarditis can be caused by a wide variety of bacteria belonging to group of Staphylococcus and Streptococcus. Streptococcus anginosus (S. anginosus) bacteremia associated with endocarditis along with colon cancer is a very rare presentation [1]. The exact pathophysiology of the association of colon cancer with anginosus is not well known.

## Case presentation

A 71-year-old woman presented to the emergency department with symptoms of dizziness, fatigue, and bright red bleeding per rectum. She has a past medical history of atrial fibrillation and hypertension. She started having six to seven bright red colored stools for about one week, unassociated with mucus. She reported concerns of constant right upper quadrant pain that started approximately two days before the presentation. She denied any nausea, vomiting, or subjective fever, and she had no recent travel or exposure to sick contacts and no history of hemorrhoids. 

On examination, she was found to be tachycardic with a heart rate of 130 beats per minute. Her blood pressure was 98/50 mmHg, and her body temperature was 100.2°F. Her oxygen saturation was 93% on room air. She appeared thin, cachectic, and had no scleral icterus. Her lungs were clear on auscultation, and her cardiovascular examination revealed no significant murmurs. 

**Figure 1:**
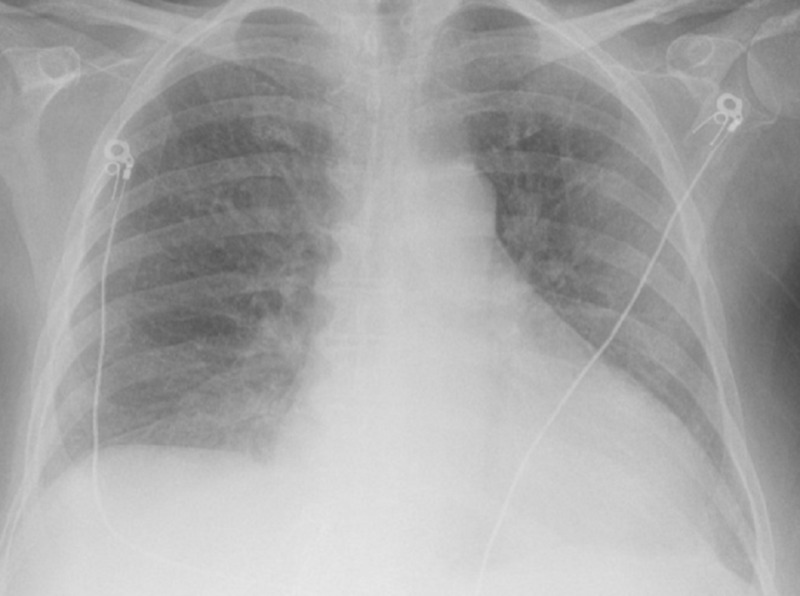
Chest X ray with no active consolidation

Her laboratory investigation showed leukocytosis with white blood cell count of 29,000 per microliter (neutrophils, 88%; lymphocytes, 3.5%; monocytes, 3.7%; eosinophils, 1.2%; basophils, 0.3%). Her hemoglobin was 9 g/dl (reference range: 11.2-15.7 g/dl), and her mean corpuscular volume was 77. Urine analysis was negative for infection. Chest X-ray did not show any active consolidation (Figure 1).

**Figure 2:**
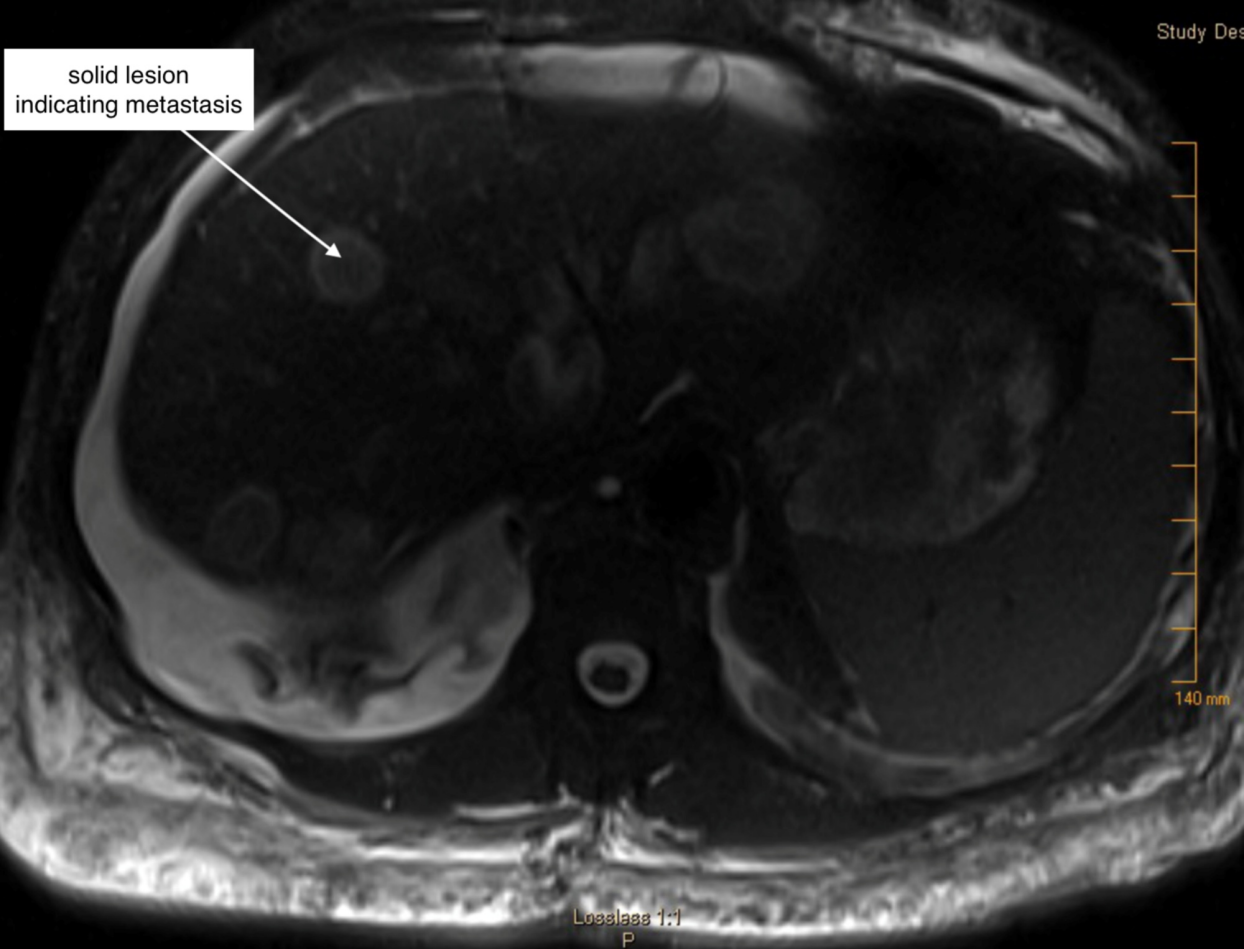
MRI abdomen showing multiple lesions in liver indicating metastasis

Her Clostridium difficile screening was negative. Right upper quadrant ultrasound showed several indeterminate hypoechoic solid masses, and both lobes of the liver had several indeterminate hypoechoic solid masses. A slight gall bladder wall thickening with no surrounding inflammatory signs was noted. MRI of the abdomen showed multiple circumscribed lesions throughout the liver compatible with metastasis (Figure 2).

The patient was started on empiric antibiotics with vancomycin and cefepime for possible infection. Her blood cultures grew S. anginosus, and her antibiotic regimen was changed to ceftriaxone.

**Figure 3:**
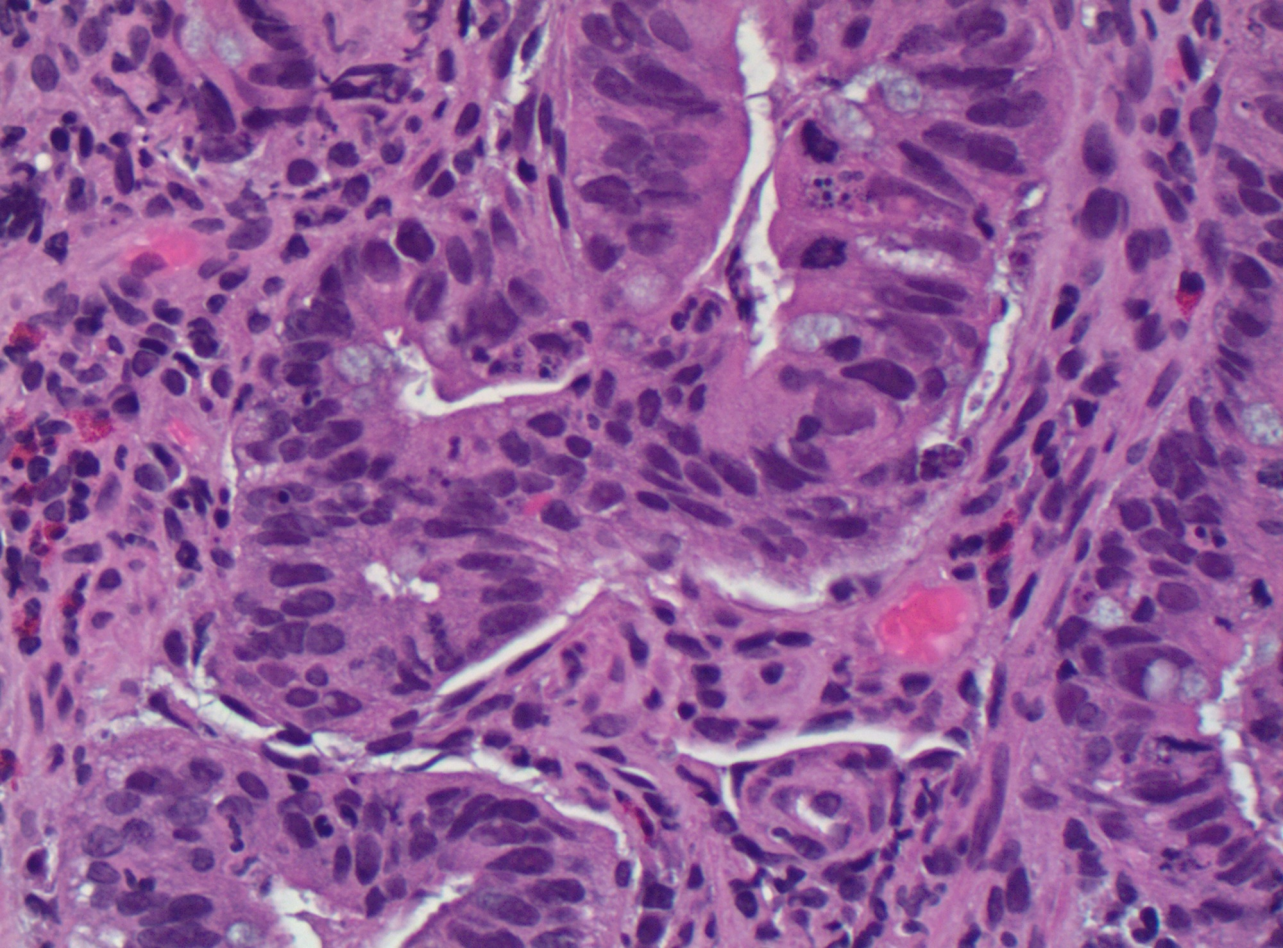
Biopsy of sigmoid mass showing moderately differentiated adenocarcinoma

As the source of the bacteremia was unknown, and because of her microcytic anemia with a recent change in bowel habits, she underwent a colonoscopy and transthoracic echocardiogram (TTE). The colonoscopy revealed a fungating infiltrative, ulcerated, partially obstructing mass in the sigmoid colon. The biopsy results are positive for moderately differentiated adenocarcinoma of the colon (Figure 3).

**Figure 4:**
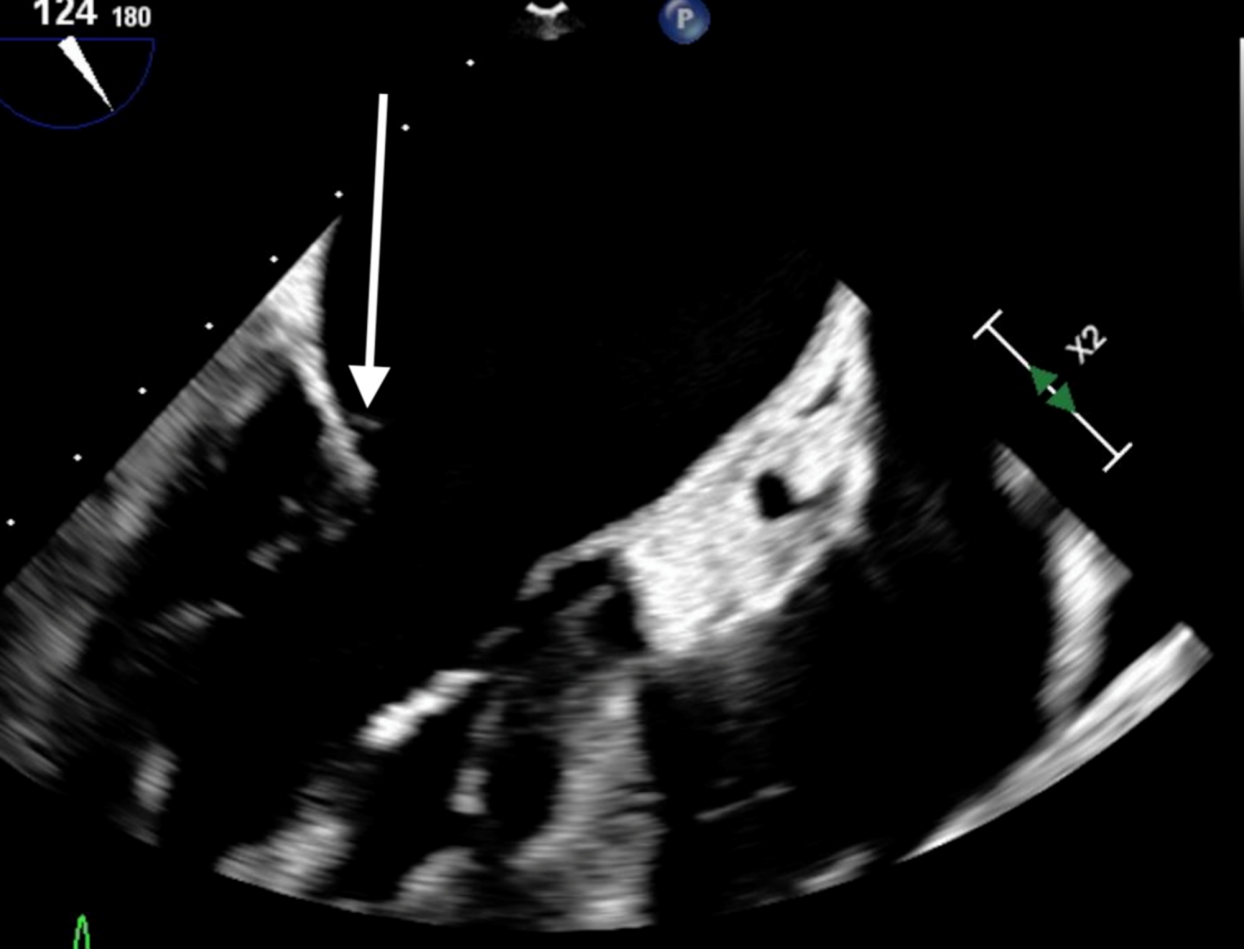
Transesophageal echocardiogram showing vegetation on mitral valve

**Figure 5:**
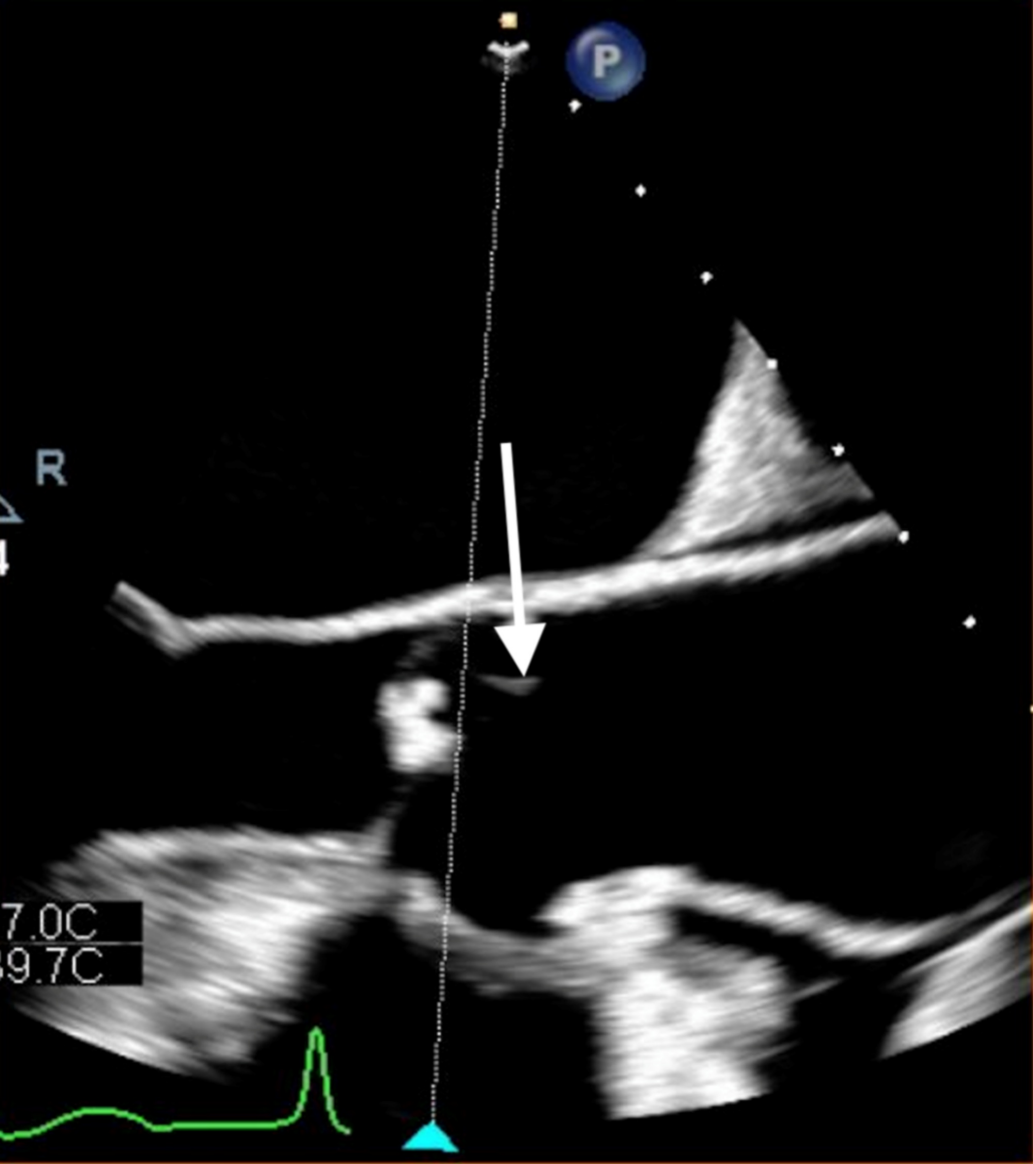
Transesophageal echocardiogram showing vegetation on the aortic valve

TTE showed thickening and possible vegetation involving the aortic and mitral valves. Subsequent transesophageal echocardiogram revealed vegetation (approximately 0.2 cm) on the aortic and mitral valves (Figures 4, 5).

She had no signs of a perivalvular abscess, and her ejection fraction was within reference range at 55% to 60%. Her care team decided to continue treatment with antibiotics for at least six weeks. Her anticoagulation for atrial fibrillation was halted due to active gastrointestinal (GI) bleeding. The patient died several days later as her family focused on comfort care measures based on the patient's wishes. 

## Discussion

S. anginosus is a normal commensal in the oral cavity and GI tract. S. anginosus is a Gram-positive and catalase-negative coccus and was formerly included in the Streptococcus milleri group. Three species (S. intermedius, S. constellatus, and S. anginosus) were included in that group. This group is unique and most commonly presents with deeply seated pyogenic infections. Common sites of involvement are the thoracic cavity, oral cavity, and the GI tract [2]. Our case is unique because our patient had both colon cancer and infective endocarditis, which are rare presentations of S. anginosus bacteremia, and only a few similar cases have been reported [1,3].

S. anginosus usually causes endocarditis in a prosthetic valve or congenital abnormal valves, but native valve endocarditis is uncommon [3]. These bacteria commonly cause extensive systemic abscess, and the pathogenesis is explained by the production of protease enzymes and hyaluronidase. There are case reports about infection with multiple liver abscesses and disseminated infection with multiple brain abscess. Our patient had multiple liver lesions, which are metastatic lesions from colon cancer rather than an abscess. It is still unknown whether S. anginosus is directly related to carcinogenesis. There may be a close association between colon cancer and infection, as cancer disrupts the normal colon mucosa, increasing the risk of disseminated infection [4]. 

However, there are no guidelines in screening for endocarditis or colon cancer in patients with anginosus group of bacteria. HANDOC scoring (HANDOC = Heart murmur or valve disease; Aetiology with the groups of S. mutans, S. bovis, S. sanguinis, or S. anginosus; Number of positive blood cultures ≥2; Duration of symptoms of ≥7 days; Only one species growing in blood cultures; and Community-acquired infection) is a validated means to determine the need for echocardiography in non-beta hemolytic Streptococcus bacteremia [5]. In this scoring system, we need to subtract a point if blood cultures are positive for the S. anginosus group. This indicates the rare association of endocarditis with anginosus. Based on these criteria, our patient score is 3 with duration, only one species in cultures and two positive blood cultures, community-acquired, and subtracting one point for positive anginosus blood cultures. It is unclear whether this scoring system applies to the anginosus group as none of the patients studied for the HANDOC score validation had anginosus endocarditis. Our patient satisfied Duke's criteria for endocarditis. Prompt diagnosis and treatment are important because a delay in treatment may increase the risk of death. Most species in the anginosus group are sensitive to penicillin and have a good response with this class of antibiotics [2].

## Conclusions

S. anginosus is a group of viridans streptococci which has a unique characteristic in forming an abscess but rarely causes endocarditis. A single positive culture should never be ignored and should prompt a laboratory workup to determine the source of infection. Early recognition and prompt initiation of antibiotics in patients with an infection can reduce the risk of poor outcomes, as most species are penicillin-sensitive. However, guidelines for colon cancer screening in a patient with S. anginosus bacteremia are not explicit. 

## References

[1] Finn T, Schattner A, Dubin I, Cohen R (2018). Streptococcus anginosus endocarditis and multiple liver abscesses in a splenectomised patient. BMJ Case Rep.

[2] Bert F, Bariou‐Lancelin M, Lambert‐Zechovsky N (1998). Clinical significance of bacteremia involving the "Streptococcus milleri" group: 51 cases and review. Clin Infect Dis.

[3] Woo PCY, Tse H, Chan KM (2004). “Streptococcus milleri” endocarditis caused by S treptococcus anginosus. Diagn Microbiol Infect Dis.

[4] Masood U, Sharma A, Lowe D, Khan R, Manocha D (2016). Colorectal cancer associated with Streptococcus anginosus bacteremia and liver abscesses. Case Rep Gastroenterol.

[5] Sunnerhagen T, Törnell A, Vikbrant M, Nilson B, Rasmussen M (2018). HANDOC: a handy score to determine the need for echocardiography in non-β-hemolytic streptococcal bacteremia. Clin Infect Dis.

